# Does fasting protect liver from ischemia and reperfusion injury?

**DOI:** 10.1590/acb384723

**Published:** 2023-10-23

**Authors:** Marcia Kiyomi Koike, Denise Frediani Barbeiro, Heraldo Possolo de Souza, Marcel Cerqueira César Machado

**Affiliations:** 1 Universidade de São Paulo – School of Medicine – Department of Clinical Medicine – São Paulo (SP) – Brazil.

**Keywords:** Liver, Ischemia, Reperfusion, Fasting, Models, Animal, Rats

## Abstract

**Purpose:**

To evaluate local and systemic effects of 24-hour fasting in liver ischemia and reperfusion injury.

**Methods:**

Twenty-one adult male Wistar rats (330–390 g) were submitted to 60 minutes of hepatic ischemia followed by 24 hours of reperfusion. Before the day of the experiment, the animals fasted, but free access to water was allowed. Two groups were constituted: Control: non-fasted, that is, feeding *ad libitum* before surgical procedure; Fasting: rats underwent previous fasting of 24 hours. Hepatic ischemia was performed using vascular clamp in hepatic pedicle. At 24 hours after liver reperfusion, blood and tissue samples were collected. To analysis, liver lobes submitted to ischemia was identified as ischemic liver and paracaval non-ischemic lobes as non-ischemic liver. We evaluated: malondialdehyde levels, hepatocellular function (alanine aminotransferase, aspartate aminotransferase activities, and both ratio), cytokines (interleukins-6, -10, and tumor necrosis factor-alpha), hepatic ischemia and reperfusion injury (histology).

**Results:**

Malondialdehyde measured in non-ischemic and ischemic liver samples, hepatocellular function and cytokines were comparable between groups. Histological findings were distinct in three regions evaluated. Microvesicular steatosis was comparable between 24-hour fasting and non-fasted control groups in periportal region of hepatic lobe. In contrast, steatosis was more pronounced in zones 2 and 3 of ischemic liver samples of fasting compared to control groups.

**Conclusions:**

These data indicates that fasting does not protect, but it can be also detrimental to liver submitted to ischemia/reperfusion damage. At that time, using long fasting before liver surgery in the real world may be contraindicated.

## Introduction

Preoperative fasting (8–12 hours) is routinely applied in elective surgeries to reduce gastric volume and acidity, helping to avoid acute respiratory obstructions, aspirative pneumonia and Mendelson’s syndrome during anesthesia. However, adverse effects of prolonged fasting include patient’s discomfort, glucose metabolic changes, and insulin resistance. To minimize these effects, several clinical studies have evaluated the reduction of fasting time or the administration of oral carbohydrate solutions in the preoperative period.

Reducing the fasting period and administering oral glucose solutions preoperatively have been shown to be effective in increasing postoperative comfort, reducing insulin resistance and postoperative stress^1^-^8^.

Experimental studies, however, have shown that fasting can have a beneficial effect in situations of hepatic^9^, renal^10^,^11^ and cardiac ischemia and reperfusion^12^,^13^. In the study by Zhan et al.^8^, 24-hour fasting was defined as short-term, while the control group underwent 6-hour fasting, and the authors observed a reduced ischemia and reperfusion damage in patients with 24-hour fasting period. Fasting protection could be explained by the reduction in lactate production and, consequently, its accumulation, in addition to the displacement of the energy production pathway for the metabolism of fatty acids, favoring the maintenance of the Krebs cycle during reperfusion.

If prolonged fasting attenuates the systemic inflammatory response and the recovery of organs such as the liver in cases of ischemia and reperfusion, it would be important in surgeries with temporary reduction in hepatic blood flow.

The aim of this study was to evaluate the local and systemic effects of 24-hour fasting in liver ischemia and reperfusion (IR) injury in rats.

## Methods

This study was designed in accordance with the Guide for the Care and Use of Laboratory Animals, published by the US National Institutes of Health, and the Guidelines of Animal Experimentation of the Universidade de São Paulo School of Medicine, São Paulo, SP, Brazil, for the care and use of laboratory animals. The experimental protocol was approved by Ethics Commission of the Universidade de São Paulo, Brazil (1772/2022). Twenty-one adult male Wistar rats weighing 330–390 g were housed in cages with a controlled 12-h light/dark cycle, receiving water *ad libitum*. Before the day of experiment, the animals fasted, but free access to water was allowed.

## Experimental design and study groups

Animals were randomized into the following experimental groups:

- Control: non-fasted rats underwent 60 minutes of hepatic ischemia followed by 24 hours of reperfusion (*n* = 11);
- Fasting: rats underwent previous fasting of 24 hours and underwent 60 minutes of hepatic ischemia followed by 24 hour of reperfusion (*n* = 10).

Rats were anesthetized with intraperitoneal ketamine (100 mg/kg) and xylazine (20 mg/kg). An upper abdominal laparotomy was performed. The hepatic pedicle to the left lateral and median hepatic lobes was encircled and occluded with a 2.5-mm microvascular clamp, inducing ischemia of about 70% of the total liver volume. The incision was closed, and after a 60-minute ischemic period, the abdomen was reopened allowing clamp removal and liver reperfusion^14^. After recovery from anesthesia, the animals were returned to their cages after full recovery. At 24 hours after liver reperfusion, rats were re-anesthetized with same intraperitoneal anesthetics solution for blood sampling via abdominal cava vein and then killed by exsanguination. To analysis, liver lobes submitted to ischemia was identified as ischemic liver and paracaval non-ischemic lobes as normal liver.

Malondialdehyde (MDA) levels in the samples were determined to obtain quantitative estimation of the membrane lipid oxidative damage. Malondialdehyde was assayed in terms of thiobarbituric acid reactive substrates. The thiobarbituric acid method was used to quantify lipid peroxidation in liver, measured as thiobarbituric acid-reactive substances. Liver tissues (100 mg/mL) were homogenized in 1.15% KCl buffer, and centrifuged at 14,000 g for 20 minutes. The supernatant was then stored at -70°C. An aliquot of the supernatant was added to a reaction mixture of 1.5 mL 0.8% thiobarbituric acid, 200 μL 8.1% (v/v) SDS, 1.5 mL 20% (v/v) acetic acid, pH 3.5, and 300 μL distilled water, and heated to 90°C for 45 min. After cooling to room temperature, the samples were cleared by centrifugation at 10,000 g for 10 min, and their absorbance was measured at 532 nm using malondialdehyde bis (dimethyl acetal) as an external standard. The quantity of lipid peroxides is reported in nmol malondialdehyde equivalents/mg protein.

The degree of hepatocellular injury was assessed by analyzing serum alanine aminotransferase (ALT) and aspartate aminotransferase (AST) activities. AST and ALT levels were assayed according to the manufacturer’s protocol (Bioclin–Quibasa, Brazil). Results are expressed as units per liter U/L.

Serum cytokines interleukin (IL)-6, IL-10 and tumor necrosis factor (TNF)-alpha were determined using a commercially enzyme-linked immunosorbent assay kit according to the manufacturer’s protocol (Mybiosource, United States of America). Results are expressed as picograms per milliliter (pg/mL).

To histologic analysis, samples of liver ischemic and nonischemic lobes were fixed in 10% buffered formalin for standard hematoxylin and eosin staining. Histologic evaluation of the liver sections was performed by a single pathologist blinded to group. The severity of histologic injury was analyzed using a modification of the scoring system proposed by Quireze et al.^15^. Features were microvesicular steatosis, coagulation necrosis, lobular and portal inflammation, and sinusoidal cellularity. Each feature was assigned a score from 0 to 3 based on its absence (0) or presence to a mild (1), moderate (2), or severe (3) degree.

### Statistical analysis

The sample calculation estimated the number of animals per group at eight, considering the standardized difference in ALT dosage^14^, power of 90% and p < 0.05. Data are expressed as mean ± standard deviation or median (interquartile range), when appropriate. After the Shapiro–Wilk’s test, the T or Mann-Whitney’s test was performed to compare control and fasting groups. It was considered significantly different when p < 0.05. GraphPad Prism 9.1 was used.

## Results

Twenty-one rats were submitted to hepatic IR Injury: 11 in control group (373 ±38 g) and 10 in the fasting group (384 ± 33 g). Mortality was similar between groups (18% control *vs*. 10% fasting, p = 0.5926).

Liver enzymes (ALT, ATL, and ALT/AST ratio) and inflammatory cytokines (Il-6, Il-10 and TNF-alpha) were comparable between groups (p > 0.05, Table 1). MDA measured in non-ischemic and ischemic liver samples were comparable between groups. In non-ischemic liver, MDA was similar: 0.98 (0.78-1.1) vs. 1.0 (0.76-1.1) nMol/mLin fasting and control groups, respectively. In ischemic liver, MDA was 0.9 (0.76-1.1) vs. 0.9 (0.8-1.1) nMol/mL in fasting and control groups, respectively.

**Table 1:** Serum liver enzymes and cytokines, and MDA in non-ischemic and ischemic liver after 24 hours of hepatic ischemia and reperfusion injury in 24-hour fasting rats compared to control (non-fasted).

Serum and liver analysis	Control (n = 9)	Fasting (n = 8)	P-value
AST (U/L)	136 (130–159)	123 (95–150)	0.2087
ALT (U/L)	226 (134–244)	160 (150–204)	0.0894
AST/ALT ratio	0.62 (0.57–0.96)	0.50 (0.67–0.76)	0.7233
MDA in non-ischemic liver (nMol/mL)	1.0 (0.76–1.1)	0.98 (0.78–1.1)	0.3715
MDA in ischemic liver (nMol/mL)	0.9 (0.8–1)	0.9 (0.76–1.1)	0.1703
IL-6 (ρg/mL)	128 (124.5–136)	124.5 (123–134)	0.4365
IL-10 (ρg/mL)	80 (71.5–82.5)	74 (72.3–83.3)	0.9808
TNF-α (ρg/mL)	75 (73–76)	74.5 (72.3–79.3)	0.5226

MDA: malondialdehyde; AST: aspartate aminotransferase; ALT: aminotransferase; IL: interleukin; TNF: tumor necrosis factor. Source: Elaborated by the authors.

**Figure 1:**
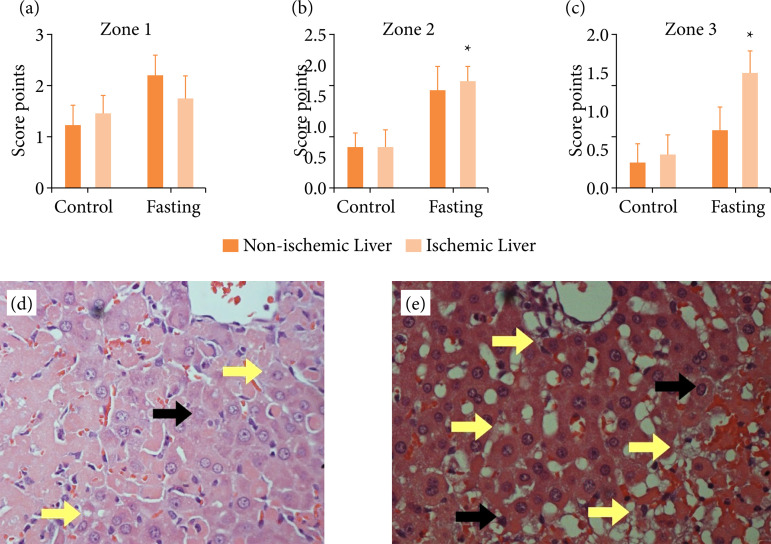
Histological assessment of hepatic ischemia reperfusion injury scoring system^15^ based on the presence and intensity of microvesicular steatosis, necrosis of hepatocyte cells in 50 randomly fields under 250x magnification. **(a)** Microvesicular steatosis was comparable between 24-hour fasting and non-fasted control groups in periportal region (Zone 1; p > 0.05) of hepatic lobe. In contrast, steatosis was more pronounced in **(b)** Zone 2 (p = 0.0014) and **(c)** Zone 3 (p = 0.0092) of ischemic and non-ischemic liver samples compared fasting to control group. **(d)** Ischemic liver in control group. **(e)** Ischemic liver in fasted group (under x40 lens). Yellow arrows indicate microvesicular steatosis and black arrows indicate necrosis.

In Table 2 and Fig. 1, histological findings were depicted. Microvesicular steatosis was comparable between 24-hour fasting and non-fasted control groups in periportal region (Fig. 1a, zone 1; p > 0.05) of hepatic lobe. In contrast, steatosis was more pronounced in zone 2 (Fig. 1b, p = 0.0014) and zone 3 (Fig. 1c, p = 0.0092) of ischemic liver samples of fasting compared to control groups. Figure 1d illustrates ischemic liver in control group, and in Fig. 1e, ischemic liver in fasting group (under x40 lens). Yellow arrows indicate microvesicular steatosis, and black arrows indicate necrosis.

**Table 2:** Histological findings (microvesicular steatosis and hepatocyte necrosis) in non-ischemic and ischemic liver after 24 hours of hepatic ischemia and reperfusion injury in 24-hour fasting rats compared to control (non-fasted).

Histological evaluation	Non-ischemic liver		Ischemic liver	
	Control (n = 9)	Fasting (n = 8)	Control (n = 9)	Fasting (n = 8)
Microvesicular steatosis (score)				
Zone 1	1.0 (0–2.5)	2.5 (2.0–3.0)	1.0 (0–2.5)	2.0 (0.25–3.0)
Zone 2	1.0 (0–1.0)	1.5 (1.0–2.75)	0 (0–1.5)	2.0 (1.0–2.0)*
Zone 3	0 (0–0.5)	0.5 (0–1.75)	0 (0–1.0)	1.0 (1.0–2.0)*
Necrosis (score)				
Zone 1	0 (0–0)	0 (0–0)	2.0 (0.5–3.0)	1.0 (0–1.75)
Zone 2	0 (0–0)	0 (0–0)	1.0 (0.5–1.5)	1.0 (0–1.75)
Zone 3	0 (0–0)	0 (0–0)	0 (0–1.0)	0.5 (0–1.75)

* p< 0.05 *vs*. control. Source: Elaborated by the authors. Source: Elaborated by the authors.

## Discussion

Previous work has shown that three-day fasting significantly decreases liver ischemic/reperfusion injury in mice and could be used in clinic in situation of liver surgery when temporary occlusion of hepatic vascular inflow (Pringle maneuver) is used to reduced blood loss^9^. The mechanism of this protection could be related to the induction of autophagy by starvation a well-known phenomenon^16^. In another study, it was shown that fasting for one, but not two or three days, protects from hepatic ischemic/reperfusion injury in mice. In this report, fasting was followed by a reduction in circulating high mobility group Box 1 (HMGB1), a potent inflammatory activity cytokine secreted by monocytes. associated with cytoplasmic HMGB-1 translocation and autophagy. Abolishing autophagy inhibits the protection induced by fasting^17^.

In another study by using perfused *ex-vivo* liver, fasting (short fasting-18 hours) failed to protect liver from I/R injury. It was suggested that high energetic charge, intracellular glycogen content and glycolytic activity protect against I/R damage^18^. However, in humans a recent study has shown that 24-hour fasting protects liver from I/R injury with attenuation of cytokine response in situation of liver surgery^8^. In this report, patients with diabetes, alcoholism and older than 75 were excluded.

In another study, hepatic cell membrane potential was evaluated in fed and fasted animals. It was observed that adenosine triphosphate (ATP) content was 40% higher and the glycogen 10 times higher in fed animals. After ischemia and reflow, membrane potential was normalized more rapidly in fed animals, what indicates that fed animals have a better recovery when compared to fasting animals^19^. From these reports, we may conclude that ischemic/reperfusion damage results from several players as autophagy, liver nutritional status as glycogen stored and inflammatory response.

In the present study, we evaluated the ischemic/reperfusion damage in 24-hour fasted compared to non-fasted animals. We did not find any benefits by fasting animals since serum cytokines, MDA levels, serum ALT and AST indicators of hepatic damage were similar in fasted and non-fasted animals (Table 1). However, histologic findings indicated an increased microvesicular steatosis in fasting liver mainly in zone 2 and zone 3 (Table 2, Fig. 1) These findings indicate a loss in the ability of the fasted liver to metabolize fatty acids and carbohydrates leading to accumulation of lipids as lipids droplets inside the hepatocyte. Similar findings have been reported in a *ex-vivo* perfused livers model^20^.

## Conclusion

We could conclude from these results that fasting does not protect, but it can be also detrimental to liver submitted to ischemia/reperfusion damage. At that time, using long fasting before liver surgery in the real world may be contraindicated.

## Data Availability

All data sets were generated or analyzed in the current study.
